# Determinants of systematic condom use among students and apprentices ages 15–24 in Benin

**DOI:** 10.1186/1471-2334-14-S2-P31

**Published:** 2014-05-23

**Authors:** Cyprien Zinsou, Joseph Inungu, Margaret Wilson

**Affiliations:** 1 Beninese Association of Social Marketing, Cotonou, Benin

## Introduction

Adolescents ages 15–24, particularly young women, continue to bear the brunt of the HIV epidemic in sub-Saharan Africa with an estimated 50% of new infections occurring in this age group. While adolescents in Benin have many sexual partners (3,4 for women and 4,5 for men) (EDS, 2012), less than 50% used condom during the last sexual encounter. The Association of Social Marketing in Benin (ABMS), in collaboration with PSI, has been implementing a youth project funded by the DUTCH embassy to reduce HIV prevalence among youth since 2012. The objective of this study is to identify the factors associated with consistent condom use in this population.

## Methods

ABMS/PSI conducted a sexual behavior survey between January-February 2013 in schools and vocational centers in the project areas to assess the sexual behavior of students and apprentices ages 15-24. Logistic regression was used to identify the factors associated with systematic condom use in this population.

## Results

A total of 4288 youths (2678 students, 1610 apprentices) were interviewed. 59.4% of them were girls and their mean age was 17,9 years. On average 17% of them (24.6% of boys and 11.7% of girls) had more than one sexual partners in the last 12 months before the survey. Of those (N=727), 46.2% used condom consistently.

The logistic regression model showed that consistent condom use among adolescents was associated with their ability to discuss about condoms (OR=2,8; p<0,001), to demonstrate how to wear condom correctly (0R=2,2 ; p<0,001), their level of knowledge about the mode of HIV transmission (0R=1,5 ; p<0,05), the fact of being a woman (OR=2,1; p<0,001) or apprentice (OR=2,2; p<0,001) and watching television regularly (OR=1,4; p<0,05).

## Conclusion

To improve consistent use of condoms among adolescents in Benin, the ABMS should take these determinants into account when designing its behavior change communication interventions.

